# Clinical efficacy of growth hormone therapy in adolescent short stature during late puberty: a prospective cohort study

**DOI:** 10.1007/s12519-026-01030-9

**Published:** 2026-04-08

**Authors:** Ke Yuan, Yong-Hua Chen, Yan-Lan Fang, Xiao-Ming Ying, Jin-Ping Chen, Wei-Dong Su, Shun-Feng Mao, Jian-Ping Zhang, Qun-Hua Shen, Jian-Fang Zhu, Chun-Lin Wang

**Affiliations:** 1 Department of Pediatrics, The First Affiliated Hospital, Zhejiang University School of Medicine, 79 Qingchun Road, Hangzhou, 310003 China; 2 Department of Pediatrics, Taizhou First People’s Hospital, Taizhou, China; 3 Department of Child Healthcare, Dongyang Maternal and Child Health Hospital, Jinhua, China; 4 Department of Pediatrics, Wenzhou Hospital of Integrated Traditional Chinese and Western Medicine, Wenzhou, China; 5 Department of Pediatrics, Affiliated Hospital of Jiaxing University, The First Hospital of Jiaxing, Jiaxing, China; 6 Department of Pediatrics, Women’s and Children’s Hospital Affiliated to Ningbo University, Ningbo, China; 7 Department of Pediatrics, Haining People’s Hospital, Jiaxing, China

Short stature affects about 3% of children worldwide [1]. Recombinant human growth hormone (rhGH) therapy has been employed to manage several pediatric growth disorders. Treatment options for increasing children’s height vary according to developmental stage. For prepubertal children [2, 3], rhGH alone is sufficient to increase height. During puberty, sex hormones have a dual effect on growth: while promoting linear growth, they also accelerate epiphyseal maturation. Therefore, in pubertal adolescents, puberty-delaying agents such as gonadotropin-releasing hormone analogs (GnRHa) and aromatase inhibitors (AIs) may be considered for use alongside rhGH. GnRHa delays puberty progression and retards skeletal maturation, but it may reduce bone mineral density and increase the risk of osteoporosis in adulthood [4]. AIs can reduce estrogen production, delay bone age (BA) advancement, and improve height outcomes in boys. However, due to lack of pediatric indications, they are not widely adopted in clinical practice [5]. Current evidence remains insufficient to clearly determine the efficacy of rhGH alone or in combination with GnRHa or AIs in treating adolescent short stature.

There exists a special group of adolescents in late puberty (Tanner stages IV–V) characterized by a declining growth velocity (GV), a BA–chronological age (CA) ≥ 1 year, and near-complete epiphyseal closure, who are at risk of short stature in adulthood. As growth potential becomes increasingly limited, therapeutic options aimed at improving final adult height (FAH) become increasingly time-sensitive. Traditionally, rhGH has been assumed to be less effective once bone maturation is advanced [6, 7]. Nevertheless, emerging evidence indicates that residual growth potential may persist even in late puberty, through the stimulation of the epiphyseal cartilage by rhGH and insulin-like growth factor 1 (IGF-1) [8, 9]. A key question is whether the therapy’s effect on FAH is clinically meaningful. A pilot study has shown that rhGH treatment in late-pubertal boys with idiopathic short stature can significantly increase FAH [10]. However, two critical limitations of this study are the absence of a control group and a small sample size. Furthermore, the safety of rhGH therapy in this population remains unclear.

This study aimed to address these critical gaps by enrolling late-pubertal adolescents with advanced BA at risk of adult short stature and to evaluate the efficacy and safety of individually optimized rhGH regimens for improving FAH in this cohort. This prospective multicenter cohort study was conducted from July 2021 to July 2024 at seven hospitals in Zhejiang Province, China. Ethical approval was obtained from the Ethics Committee of the First Affiliated Hospital of Zhejiang University School of Medicine (Expedition review No. 642 in 2021). The study adhered strictly to the principles of the Declaration of Helsinki. Written informed consent was obtained from all participants (or their legal guardian for children under 16 years of age). This study was registered in Chinese Clinical Trial Registry (ChiCTR2100043800). The inclusion criteria were as follows: (1) BA ≥ 13 years for girls and ≥ 14 years for boys, with BA − CA ≥ 1 year; (2) height standard deviation score (SDS) ≤  − 2 SD, or height between − 1 and − 2 SD with advanced BA, or height SDS for BA (HtSDS_BA_) ≤  − 2 SD, or predicted adult height (PAH) less than mid-parental target height minus 1 SD; (3) GV < 6.0 cm per year; (4) Tanner stage IV–V. The exclusion criteria included individuals with epiphyseal closure, diagnosed endocrine disorders such as growth hormone deficiency, other systemic conditions such as skeletal dysplasia, known hypersensitivity to rhGH, and participation in other growth-related drug trials within the last three months.

Participants were scheduled to be enrolled and allocated in a 2:1 ratio to either the rhGH group or the control group. Lifestyle interventions were provided as standard background care across both cohorts. The initial dosage of daily rhGH was set at 0.15–0.18 IU/kg/d for girls with a BA of 13–13.5 years and boys with a BA of 14–15.5 years, and 0.2 IU/kg/d for girls with a BA ≥ 13.5 years and boys with a BA ≥ 15.5 years. Dosage was adjusted quarterly: increased by 20% for a 3-month height increment < 1.5 cm, and reduced by 20% if IGF-1 >  + 2 SD. The maximum dosage was 0.2 IU/kg/d. FAH indications: 3-month height gains < 0.5 cm after dose escalation.

Baseline data comprised demographics, anthropometrics, and laboratory investigations. BA was assessed by the G–P method [11]. PAH was calculated using the Bayley–Pinneau method [12]. The SDS of height was expressed as HtSDS_BA_ for BA and as HtSDS for CA. HtSDS was calculated using 2005 national survey data [13]. Target height (TH) was defined as [father’s height (cm) + mother’s height (cm) ± 13]/2. FAH was defined as yearly GV < 2 cm/year. IGF-1 levels were measured centrally at a core laboratory. The following indicators were used to assess efficacy: (1) difference between FAHSDS and PAHSDS; (2) difference between FAHSDS and THSDS; (3) FAHSDS; and (4) difference between FAHSDS and height SDS at baseline (HtSDS). Efficacy outcomes will be reported by gender. Safety profile indicators included the incidence of adverse events (AEs) and serious AEs (SAEs).

Statistical analyses were performed with R (version 4.1.2). Normality was assessed by Shapiro–Wilk tests (n ≤ 50). Continuous variables were described as mean ± SD (normal) or median (IQR) (non-normal), analyzed by paired t-test/ANOVA or Kruskal–Wallis H test, respectively. Categorical variables were presented as frequencies (percentages) with chi-square test. Multivariate analysis used multiple linear regression or stratified analysis, and two-sided P < 0.05 was deemed statistically significant. Given potential baseline imbalance, an ANCOVA model was used to compare continuous outcomes between groups, adjusted for gender, CA, and height SDS.

A total of 40 participants were enrolled, of whom 39 who achieved FAH were included in the analysis (Fig. S1). The 27 males had a median age of 14 years (13.00, 14.40), BA-CA (year) of 1.79 (1.13, 2.53). Median PAHSDS was − 1.90 (− 2.17, − 1.22), and PAHSDS–THSDS was − 1.50 (− 1.75, − 1.23). The 12 females had a median age of 12.00 years (11.45, 12.85), BA-CA (year) of 1.95 (1.44, 2.56). Median PAHSDS was − 2.10 (− 2.60, − 1.58), and PAHSDS–THSDS was − 1.48 (− 1.61, − 1.07) (Table 1). CA and TH were higher in males than in females (Table S1). There were 27 cases in the rhGH group and 12 in the control group. The rhGH group had a significantly higher proportion of males than the control group (81.50% vs 41.60%). Both groups were comparable in terms of CA, BA, HtSDS, and IGF-1 levels (all *P* > 0.05). The median treatment duration in the rhGH group was 9.00 months (6.00, 12.00). The initial dose of rhGH was 0.18 ± 0.01 IU/kg/day, and the average dose throughout the treatment course was 0.19 ± 0.01 IU/kg/day (Table 1).

**Table 1:** Baseline auxological and clinical characteristics of the patients

Characteristics	Overall (*N* = 39)	By group			By gender	
		Control (*N* = 12)	rhGH (*N* = 27)	*P*	Female (*N* = 12)	Male (*N* = 27)
Male	27 (69.23%)	5 (41.67%)	22 (81.48%)	0.023	–	–
CA, y	13.00 (12.31, 14.16)	13.00 (12.53, 14.13)	13.11 (12.31, 14.16)	0.795	12.00 (11.45, 12.85)	14.00 (13.00, 14.40)
Height (B), cm	158.20 (150.85, 162.00)	150.85 (148.23, 159.50)	159.00 (157.40, 162.00)	0.061	147.50 (145.38, 149.38)	159.50 (158.15, 163.20)
HtSDS	− 0.54 (− 1.31, − 0.19)	− 1.03 (− 1.49, − 0.39)	− 0.46 (− 1.08, − 0.16)	0.11	− 0.79 (− 1.25, − 0.30)	− 0.54 (− 1.31, 0.06)
BMI SDS	0.38 (− 0.31, 1.11)	0.14 (− 0.43, 1.50)	0.39 (− 0.15, 0.97)	0.893	0.51 (− 0.07, 0.99)	0.38 (− 0.31, 1.11)
IGF-1 SDS	− 1.16 (− 1.87, − 0.37)	− 1.41 (− 1.87, − 1.23)	− 0.85 (− 1.58, − 0.32)	0.109	− 1.13 (− 1.52, − 0.37)	− 1.19 (− 1.88, − 0.44)
IGF-1/IGFBP-3 molar ratio	0.21 (0.18, 0.26)	0.19 (0.17, 0.27)	0.22 (0.19, 0.25)	0.454	0.26 (0.21, 0.29)	0.21 (0.18, 0.24)
BA, y	15.25 (14.25, 15.88)	15.20 (13.50, 15.63)	15.25 (14.88, 15.88)	0.417	13.63 (13.50, 14.13)	15.50 (15.25, 16.00)
BA − CA, y	1.89 (1.23, 2.55)	1.65 (1.19, 2.26)	1.90 (1.30, 2.82)	0.361	1.95 (1.44, 2.56)	1.79 (1.13, 2.53)
HtSDS_BA_	− 1.79 (− 2.05, − 1.21)	− 1.66 (− 2.37, − 1.18)	− 1.79 (− 1.97, − 1.31)	0.48	− 1.98 (− 2.34, − 1.35)	− 1.71 (− 1.94, − 1.18)
HV, cm/y	4.00 (2.90, 5.00)	4.00 (3.25, 4.88)	4.00 (2.80, 5.00)	0.919	4.00 (2.85, 4.25)	4.25 (3.40, 5.13)
PAH, cm	159.67 (152.83, 163.69)	153.22 (148.95, 160.80)	160.48 (158.49, 164.15)	**0.049**	149.32 (146.62, 152.12)	161.18 (159.51, 165.26)
PAHSDS	− 1.93 (− 2.21, − 1.36)	− 1.82 (− 2.60, − 1.38)	− 1.93 (− 2.20, − 1.37)	0.558	− 2.10 (− 2.60, − 1.58)	− 1.90 (− 2.17, − 1.22)
TH, cm	169.50 (162.00, 173.00)	162.00 (155.75, 170.88)	170.00 (166.75, 173.25)	**0.041**	156.00 (153.44, 161.38)	172.00 (169.25, 173.50)
THSDS	− 0.36 (− 0.85, 0.14)	− 0.28 (− 1.22, 0.13)	− 0.36 (− 0.77, 0.14)	0.484	− 0.85 (− 1.33, 0.15)	− 0.11 (− 0.56, 0.14)
PAH-TH, cm	− 8.49 (− 10.03, − 6.74)	− 8.24 (− 8.91, − 7.23)	− 8.66 (− 10.32, − 6.74)	0.408	− 7.90 (− 8.60, − 5.73)	− 9.08 (− 10.61, − 7.47)
PAHSDS-THSDS	− 1.50 (− 1.71, − 1.15)	− 1.40 (− 1.67, − 1.26)	− 1.54 (− 1.71, − 1.11)	0.799	− 1.48 (− 1.61, − 1.07)	− 1.50 (− 1.75, − 1.23)
HtSDS_BA_-THSDS	− 1.29 (− 1.52, − 1.06)	− 1.27 (− 1.40, − 1.12)	− 1.29 (− 1.57, − 1.04)	0.538	− 1.20 (− 1.39, − 0.85)	− 1.39 (− 1.62, − 1.16)
Treatment duration, (mon)	–	–	9.00 (6.00, 12.00)	–	9.00 (6.00, 9.00)	9.00 (6.00, 12.00)
Follow-up duration, (mon)	15.00 (12.00, 21.00)	15.00 (12.00, 19.50)	15.00 (10.50, 21.00)	0.794	12.00 (11.25, 15.00)	18.00 (12.00, 24.00)
Initial rhGH dose, IU/kg.d (mean ± SD)	–	–	0.18 ± 0.01	–	0.18 ± 0.00	0.18 ± 0.02
Initial rhGH dose, IU/kg.d [median (IQR)]	–	–	0.18 (0.17, 0.20)	–	0.18 (0.18, 0.18)	0.18 (0.17, 0.20)

Values are expressed as median (IQR) or mean ± SD

*CA* chronological age, *Height (B)* height at baseline, *SD* standard deviation, *SDS* standard deviation score, *Ht* height, *BMI* body mass index, *IGF-1* insulin-like growth factor 1, *IGFBP-3* insulin-like growth factor binding protein 3, *BA* bone age, *BA-CA* the difference between bone age and chronological age, *HtSDS* standard deviation score for height by chronological age, *HtSDS*_*BA*_ standard deviation score for height by bone age, *HV* height velocity, *PAH* predicted adult height for bone age, *TH* target height, *rhGH* recombinant human growth hormone, *PAHSDS-THSDS* difference between predicted adult height SDS and target height SDS, represents the deficit of predicted final adult height compared with genetic height, *HtSDS*_*BA*_*-THSDS* difference between bone age-specific baseline height SDS and target height SDS, represents the deficit compared with genetic height at baseline. Bold character indicates *P* values of significance

In male patients, both groups achieved FAH exceeding PAH. The gain in height (FAH SDS-PAH SDS) was significantly greater in the rhGH group [1.39 (0.97, 1.76) vs 0.33 (0.24, 0.75), *P* = 0.002], as was height gain from baseline (FAH-Height (B) [7.20 (4.15, 8.53) vs 2.00 (0.50, 4.20), *P* = 0.01] (Table 2). FAH approached TH in both groups, but the difference (FAHSDS-THSDS) was significantly smaller in the rhGH group [− 0.21 (− 0.63, 0.51) vs − 0.96 (− 1.19, − 0.93), *P* = 0.005]. The proportion of patients reaching the genetic height (FAHSDS-THSDS ≥  − 1) was also higher in the rhGH group (90.91% vs 60.00%). In female patients, FAH exceeded PAH in both groups. Notably, the rhGH group showed significantly greater improvement [1.61 (1.43, 1.83) vs 0.81 (0.60, 0.97), *P* = 0.048], consistent with male patients. Although differences in other indicators did not reach statistical significance, all values favored the rhGH group. Stratified by treatment duration, the rhGH group was divided into long-term (≥ 12 months, *n* = 8) and short-term (< 12 months, *n* = 19) subgroups. The long-term subgroup achieved significantly greater height improvement in two measures: FAHSDS-THSDS [0.47 (0.37, 0.69) vs − 0.32 (− 0.70, 0.21), *P*= 0.015], and FAHSDS-PAHSDS [1.80 (1.52, 2.07) vs 1.27 (0.86, 1.68), *P* = 0.015] (Table S2). These results indicate that treatment for ≥ 12 months correlates with improved height outcomes in late-pubertal adolescents. A multivariate regression analysis was conducted. The results showed that the model consistency was poor, presumably due to the small sample size. However, the treatment duration was a positive factor affecting each outcome indicator (Table S3).

**Table 2:** Comparison of outcomes between gender groups

Characteristics	Female			Male		
	Control (*N* = 7)	rhGH (*N* = 5)	*P*	Control (*N* = 5)	rhGH (*N* = 22)	*P*
Age at FAH, y	13.50 (13.25, 13.98)	12.30 (12.00, 12.30)		16.00 (15.50, 16.25)	15.22 (14.09, 15.75)	–
FAH, cm	150.00 (148.45, 153.50)	150.10 (150.00, 153.50)	0.744	165.00 (159.50, 166.70)	168.10 (162.50, 170.00)	0.159
FAH-Height (B), cm	2.80 (0.25, 4.70)	3.50 (3.40, 5.00)	0.29	2.00 (0.50, 4.20)	7.20 (4.15, 8.53)	**0.01**
FAHSDS	− 1.51 (− 1.98, − 0.62)	− 0.57 (− 0.63, − 0.35)	0.149	− 1.06 (− 1.90, − 0.58)	− 0.47 (− 0.92, − 0.14)	0.086
FAHSDS-HtSDS	− 0.27 (− 0.58, 0.12)	0.00 (− 0.01, 0.15)	0.432	− 0.50 (− 0.59, − 0.42)	0.03 (− 0.25, 0.23)	0.099
FAHSDS-HtSDS_BA_	0.60 (0.40, 0.70)	1.39 (1.17, 1.49)	0.03	0.21 (0.10, 0.75)	1.26 (0.86, 1.62)	**0.005**
FAH-TH, cm	− 6.50 (− 7.00, − 5.25)	− 6.00 (− 7.30, − 4.00)	> 0.9	− 8.70 (− 10.30, − 7.00)	− 4.65 (− 6.23, − 2.14)	**0.025**
FAHSDS-THSDS	− 0.47 (− 0.97, − 0.01)	0.28 (− 0.72, 0.48)	0.202	− 0.96 (− 1.19, − 0.93)	− 0.21 (− 0.63, 0.51)	**0.005**
FAHSDS-THSDS ≥ − 1	5 (71.43%)	5 (100.00%)	–	3 (60.00%)	20 (90.91%)	–
FAH-PAH, cm	− 0.08 (− 0.69, 2.14)	2.41 (1.07, 2.54)	0.149	0.80 (− 0.57, 1.26)	5.21 (2.78, 6.97)	**0.008**
FAHSDS-PAHSDS	0.81 (0.60, 0.97)	1.61 (1.43, 1.83)	0.048	0.33 (0.24, 0.75)	1.39 (0.97, 1.76)	**0.002**
FAHSDS-PAHSDS ≥ 1	2 (28.57%)	4 (80.00%)	–	0 (0.00%)	16 (72.73%)	–

Values are expressed as median (IQR)

*FAH* final adult height, *SDS* standard deviation score, *Height (B)* height at baseline, *BA* bone age, *HtSDS* height standard deviation score at baseline, *FAHSDS-HtSDS*_*BA*_ The difference between final adult height SDS and baseline bone age-specific height SDS, *PAH* predicted adult height at baseline, *FAHSDS-PAHSDS* the difference between final adult height SDS and predicted adult height SDS at baseline, representing the benefit compared to the baseline predicted height, *FAHSDS-THSDS* the difference between final adult height SDS and target height SDS. Bold characters indicates *P* values of significance

One patient reported mild joint pain after six months of rhGH treatment, which resolved spontaneously within two days and was considered possibly treatment-related by the investigator. No SAEs were observed. During the rhGH therapy, IGF-1 levels remained within the range of − 2 SD to + 2 SD (Fig. S2). A few participants had minor, clinically insignificant abnormalities in fasting glucose, insulin, and thyroid hormone, without requiring intervention. Overall, rhGH therapy was well tolerated, with no discontinuations due to safety concerns (Table 3).

**Table 3:** Safety indicators during PEG-rhGH treatment and follow-up period

Indicators	Duration, mon								
	0	3	6	9	12	15	18	21	24
FPG, mmol/L	5.06 ± 0.33 (*n* = 26)	5.01 ± 0.41 (*n* = 23)	5.05 ± 0.43 (*n* = 18)	5.16 ± 0.52 (*n* =12)	5.13 ± 0.32 (*n* = 7)	5.27 ± 0.44 (*n* = 5)	5.20 ± 0.14 (*n* = 3)	5.13 ± 0.11 (*n* = 2)	5.41 ± NA (*n* = 1)
HbA1c (%)	5.41 ± 0.57 (*n* = 25)	5.43 ± 0.45 (*n* = 13)	5.33 ± 0.44 (*n* = 16)	5.42 ± 0.23 (*n* = 10)	5.78 ± 0.40 (*n* = 6)	5.58 ± 0.23 (*n* = 6)	5.65 ± 0.07 (*n* = 2)	5.80 ± NA (*n* = 1)	5.90 ± NA (*n* = 1)
FINS, μIU/L	10.02 ± 6.13 (*n* = 25)	9.08 ± 4.14 (*n* = 16)	10.64 ± 6.04 (*n* = 19)	8.67 ± 4.18 (*n* = 12)	14.10 ± 7.08 (*n* = 7)	12.86 ± 6.91 (*n* = 7)	13.90 ± NA (*n* = 1)	21.75 ± 11.38 (*n* = 2)	22.80 ± NA (*n* = 1)
TSH, mIU/L	2.33 ± 2.54 (*n* = 27)	1.82 ± 1.40 (*n* = 24)	1.52 ± 0.89 (*n* = 20)	1.96 ± 1.16 (*n* = 15)	2.07 ± 1.38 (*n* = 7)	1.97 ± 1.28 (*n* = 7)	2.20 ± 1.22 (*n* = 3)	2.92 ± 2.02 (*n* = 2)	1.52 ± NA (*n* = 1)
T3, nmol/L	1.78 ± 0.48 (*n* = 27)	1.99 ± 0.36 (*n* = 23)	1.92 ± 0.34 (*n* = 20)	1.74 ± 0.32 (*n* = 15)	1.76 ± 0.36 (*n* = 7)	1.68 ± 0.37 (*n* = 7)	1.84 ± 0.66 (*n* = 3)	1.64 ± 0.02 (*n* = 2)	1.85 ± NA (*n* = 1)
T4, nmol/L	91.71 ± 19.66 (*n* = 27)	87.22 ± 15.76 (*n* = 23)	89.37 ± 15.27 (*n* = 20)	87.29 ± 23.44 (*n* = 15)	100.86 ± 23.98 (*n* = 7)	94.79 ± 12.34 (*n* = 7)	94.83 ± 15.08 (*n* = 3)	118.45 ± 0.77 (*n* = 2)	101.17 ± NA (*n* = 1)
FT3, pmol/L	5.04 ± 1.01 (*n* = 27)	5.27 ± 1.30 (*n* = 22)	5.08 ± 0.75 (*n* = 20)	4.79 ± 0.85 (*n* = 15)	5.08 ± 0.41 (*n* = 7)	4.92 ± 0.65 (*n* = 7)	5.42 ± 1.24 (*n* = 3)	4.16 ± 0.79 (*n* = 2)	4.82 ± NA (*n* = 1)
FT4, pmol/L	13.03 ± 1.23 (*n *= 27)	12.25 ± 1.66 (*n* = 23)	12.76 ± 1.44 (*n* = 20)	12.25 ± 2.35 (*n* = 15)	12.64 ± 2.28 (*n* = 7)	12.90 ± 2.00 (*n* = 7)	11.29 ± 1.02 (*n* = 3)	12.80 ± 3.90 (*n* = 2)	12.45 ± NA (*n* = 1)

Data are presented as mean ± SD

*FPG* fasting plasma glucose, *HbA1c* Hemoglobin A1c, *FINS* fasting insulin, *TSH* thyroid-stimulating hormone, *T3* triiodothyronine, *T4* thyroxine, *FT3* free triiodothyronine, *FT4* free thyroxine, *NA* not available

This study showed that while late-pubertal adolescents with advanced BA (predicted short stature or inability to attain genetic TH) maintained some spontaneous growth potential, their natural height catch-up was limited. RhGH therapy significantly enhanced height outcomes, leading to FAH closer to genetic TH and substantially higher than PAH. These results confirm the persistent therapeutic value of rhGH in patients approaching epiphyseal closure.

We studied a clinically ambiguous cohort with normal height and pubertal onset, but accelerated bone maturation leading to a risk of short adult height or failure to achieve optimal genetic height. Retrospective evaluation of pubertal tempo was unavailable, so we could not verify whether these cases represented the rare rapid-tempo puberty reported previously [14].

Although short stature can impair psychosocial outcomes in adolescents, uncertain efficacy and financial costs create a clinical dilemma requiring careful pretreatment counseling. In this study, treatment was initiated following shared decision-making between clinicians and patients regarding risks and benefits [15]. These findings challenge the conventional assumption that rhGH treatment initiated in late puberty yields minimal benefit. Earlier studies focused on rhGH use in prepubertal and early pubertal stages [2, 3], however, the present results show that even adolescents in late puberty can derive growth benefit from rhGH therapy. This aligns with emerging biological and clinical evidence that the growth plate may retain responsiveness to rhGH/IGF-1 signaling beyond previous expectations [8, 16].

A key finding of this study is a positive correlation between rhGH treatment duration and FAH gain. Patients treated for more than 12 months achieved significantly better outcomes. This highlights that even near epiphyseal closure, standardized therapy with individualized dosing still significantly improves therapeutic efficacy, underscoring the need for routine growth monitoring in pubertal adolescents. RhGH should be initiated promptly when GV falls below normal ranges to optimize FAH. RhGH therapy was well tolerated, with only mild, transient elevations in IGF-1 and no SAEs. Despite concerns that IGF-1 elevation may predispose patients to metabolic disturbances, no clinically significant issues including insulin resistance or glucose intolerance were observed during treatment. These findings reinforce rhGH therapy safety in pediatric patients, even in late puberty [17].

The present study has several limitations. The non-randomized, open-label design may have introduced potential biases. Additionally, the final sample size was lower than planned, with gender imbalance observed across groups. To improve robustness, we adopted several strategies: standardized height measurements and gender-stratified analyses. We further performed ANCOVA adjusted for baseline gender, which validated the primary finding of a significant therapeutic advantage in the rhGH group (Table S4). Although the sample size was below the planned target, post hoc power analysis for the primary endpoint (FAH SDS-PAH SDS) showed 98.0% power at α = 0.05, confirming adequate statistical power to detect between-group differences. However, the small sample may have impaired estimation precision. Our real-world findings are thus hypothesis-generating which require validation in larger studies.

In conclusion, this study demonstrates that rhGH monotherapy is effective and safe in late-pubertal adolescents. Individualized treatment yields meaningful height gains despite impending epiphyseal closure, and earlier intervention predicts better outcomes, with favorable tolerability. Late puberty should not be considered a lost intervention opportunity; with appropriate patient selection and tailored treatment, rhGH therapy remains viable for linear growth in this group. Further research is warranted to refine rhGH’s optimal timing and long-term safety in adolescents nearing growth cessation.

## Supplementary Information

Below is the link to the electronic supplementary material.

Supplementary file 1 (TIFF 67 KB)

Supplementary file 2 (TIFF 212 KB)

Supplementary file 3 (DOCX 39 KB)

## Data Availability

The data that support the findings of this study are available from the authors, but restrictions apply to their availability. These data were used under license from the Office of Scientific Research at The First Affiliated Hospital, Zhejiang University School of Medicine for the current study and are therefore not publicly accessible. However, the data can be made available by the authors upon reasonable request and with permission from the institution.
