# Supplementary figures and images for: Clinical efficacy of growth hormone therapy in adolescent short stature during late puberty: a prospective cohort study
Source: World J Pediatr. 2026 Apr 8;22(4):477–82. doi: 10.1007/s12519-026-01030-9 (PMC13221340; doi:10.1007/s12519-026-01030-9)

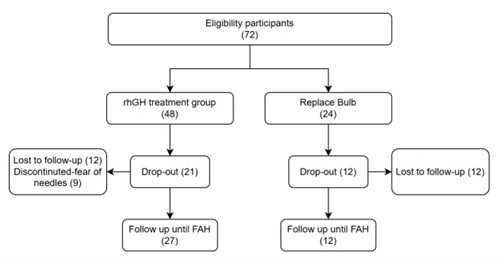

Supplement: Supplementary file 1 — (TIFF 67 KB) [file 12519_2026_1030_MOESM1_ESM.tiff]

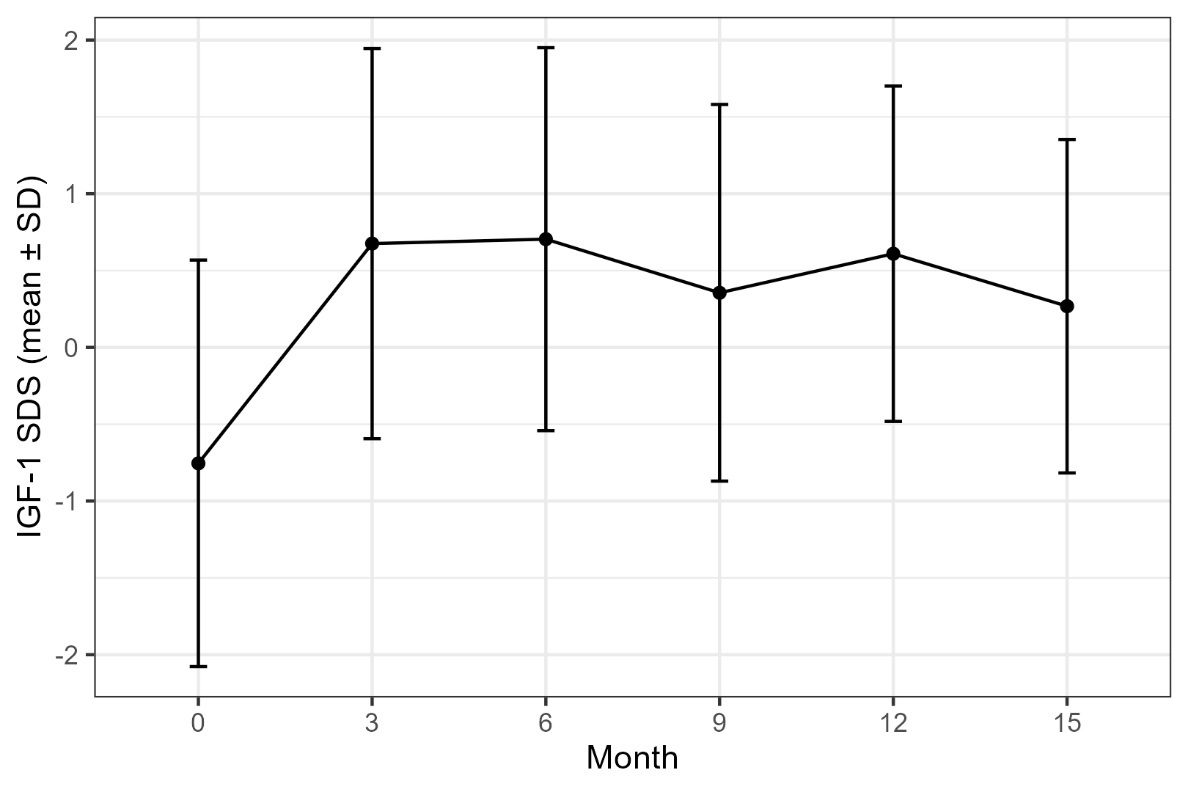

Supplement: Supplementary file 2 — (TIFF 212 KB) [file 12519_2026_1030_MOESM2_ESM.tiff]
