# Supplementary material for: Clinical efficacy of growth hormone therapy in adolescent short stature during late puberty: a prospective cohort study
Source: World J Pediatr. 2026 Apr 8;22(4):477–82. doi: 10.1007/s12519-026-01030-9 (PMC13221340; doi:10.1007/s12519-026-01030-9)
Supplement: Supplementary file 3 — (DOCX 39 KB) [file 12519_2026_1030_MOESM3_ESM.docx]

**Supplementary Materials**

**Figure legend**

**Fig. S1. Flowchart of patient screening**

**Fig. S2. Changes in IGF-1 SDS in the rhGH group**

*IGF-1* insulin-like growth factor 1, *SD* standard deviation, *SDS* standard deviation score

**Table S1. Baseline characteristics stratified by gender and treatment group**

| Characteristic |  | Control | | | rhGH | | |
| --- | --- | --- | --- | --- | --- | --- | --- |
|  | Overall N = 39 | Female N = 7 | Male N = 5 | *P*-value | Female N = 5 | Male N = 22 | *P*-value |
| Male | 27 (69.23%) |  |  |  |  |  |  |
| CA (year) | 13.00 (12.31, 14.16) | 12.70 (12.00, 13.00) | 14.50 (14.00, 14.70) | 0.005 | 11.60 (11.00, 12.00) | 14.00 (13.00, 14.28) | 0.002 |
| Height (B) (cm) | 158.20 (150.85, 162.00) | 149.00 (145.10, 149.65) | 162.50 (158.50, 163.00) | 0.003 | 146.50 (145.70, 148.50) | 159.50 (158.13, 163.05) | 0.001 |
| HtSDS | −0.54 (−1.31, −0.19) | −1.22 (−1.54, −0.57) | −0.85 (−1.41, −0.40) | 1 | −0.34 (−0.78, −0.18) | −0.50 (−1.23, 0.19) | 0.685 |
| BMI SDS | 0.38 (−0.31, 1.11) | 0.80 (0.14, 1.70) | −0.37 (−0.59, −0.20) | 0.149 | 0.23 (−0.47, 0.84) | 0.44 (0.01, 1.14) | 0.344 |
| IGF-1 SDS | −1.16 (−1.87, −0.37) | −1.41 (−1.52, −1.13) | −1.64 (−1.97, −1.36) | 0.73 | −0.40 (−1.01, −0.35) | −1.01 (−1.58, 0.02) | 0.82 |
| IGF-1/IGFBP-3 | 0.21 (0.18, 0.26) | 0.28 (0.22, 0.30) | 0.18 (0.16, 0.18) | 0.095 | 0.25 (0.20, 0.28) | 0.22 (0.19, 0.25) | 0.447 |
| BA (year) | 15.25 (14.25, 15.88) | 13.50 (13.38, 14.75) | 15.50 (15.50, 16.50) | 0.049 | 13.75 (13.50, 14.00) | 15.50 (15.25, 16.00) | 0.001 |
| BA−CA (year) | 1.89 (1.23, 2.55) | 2.00 (1.03, 2.38) | 1.50 (1.50, 1.79) | 0.871 | 1.90 (1.70, 2.75) | 1.95 (1.09, 2.82) | 0.662 |
| HtSDSBA | −1.79 (−2.05, −1.21) | −2.06 (−2.45, −1.13) | −1.33 (−1.98, −1.27) | 1 | −1.91 (−2.05, −1.57) | −1.75 (−1.90, −1.05) | 0.314 |
| HV (cm/year) | 4.00 (2.90, 5.00) | 4.00 (3.50, 4.75) | 4.00 (2.50, 5.00) | 1 | 3.35 (2.53, 4.00) | 4.50 (3.60, 5.10) | 0.19 |
| PAH (cm) | 159.67 (152.83, 163.69) | 149.70 (146.57, 152.52) | 164.20 (159.67, 164.65) | 0.005 | 148.93 (147.74, 150.96) | 161.07 (159.51, 165.34) |  |
| PAHSDS | −1.93 (−2.21, −1.36) | −2.03 (−2.61, −1.50) | −1.40 (−2.15, −1.32) | 0.639 | −2.17 (−2.39, −1.79) | −1.91 (−2.17, −1.21) | 0.232 |
| TH (cm) | 169.50 (162.00, 173.00) | 156.00 (153.00, 161.00) | 172.00 (170.50, 173.00) | 0.015 | 156.00 (154.00, 161.00) | 171.38 (169.13, 173.50) | 0.001 |
| THSDS | −0.36 (−0.85, 0.14) | −0.85 (−1.41, 0.08) | −0.11 (−0.36, 0.06) | 0.515 | −0.85 (−1.22, 0.08) | −0.21 (−0.58, 0.14) | 0.333 |
| PAH−TH (cm) | −8.49 (−10.03, −6.74) | −7.54 (−8.64, −6.02) | −8.35 (−10.83, −8.13) | 0.202 | −8.26 (−8.41, −5.68) | −9.32 (−10.35, −6.91) | 0.314 |
| PAHSDS−THSDS | −1.50 (−1.71, −1.15) | −1.41 (−1.62, −1.13) | −1.38 (−1.79, −1.34) | 0.432 | −1.54 (−1.57, −1.06) | −1.54 (−1.71, −1.14) | 0.832 |
| HtSDS_BA_−THSDS | −1.29 (−1.52, −1.06) | −1.21 (−1.32, −0.93) | −1.39 (−1.63, −1.29) | 0.106 | −1.20 (−1.48, −0.85) | −1.37 (−1.59, −1.12) | 0.524 |
| Follow-up duration (month) | 15.00 (12.00, 21.00) | 15.00 (12.00, 16.50) | 18.00 (12.00, 24.00) | 0.406 | 12.00 (9.00, 12.00) | 16.50 (12.75, 23.25) | 0.044 |
| Treatment duration (month) |  |  |  |  | 9.00 (6.00, 9.00) | 9.00 (6.00, 12.00) | 0.824 |
| Initial rhGH dose (IU/kg), Mean ± SD |  |  |  |  | 0.18 ± 0.00 | 0.18 ± 0.02 | 0.222 |
| Initial rhGH dose (IU/kg), Median (IQR) |  |  |  |  | 0.18 (0.18, 0.18) | 0.18 (0.17, 0.20) | 0.414 |
| Note: Dose is expressed as mean ± SD and median (IQR) while other values are expressed only as median (IQR).  *CA* chronological age, *Height (B)* height at baseline, *SD* standard deviation, *SDS* standard deviation score, *Ht* height, *BMI* body mass index, *IGF-1* insulin-like growth factor 1, *IGFBP-3* insulin-like growth factor binding protein 3, *BA* bone age, *BA−CA* the difference between bone age and chronological age, *HtSDS* standard deviation score for height by chronological age, *HtSDS_BA_* standard deviation score for height by bone age, *HV* height velocity, *PAH* predicted adult height for bone age, *TH* target height, *rhGH* recombinant human growth hormone, *PAHSDS−THSDS* difference between predicted adult height SDS and target height SDS,represents the deficit of predicted final adult height compared with genetic height, *HtSDS_BA_−THSDS* difference between bone age-specific baseline height SDS and target height SDS,represents the deficit compared with genetic height at baseline | | | | | | | |

**Table S2. Stratified analysis by duration of treatment (cutoff value 12 months)**

| Characteristic | < 12 months | ≥ 12 months | *P*-value |
| --- | --- | --- | --- |
|  | N = 19 | N = 8 |  |
| FAH (cm) | 162.50 (160.30, 166.50) | 169.85 (168.80, 174.40) | 0.059 |
| FAHSDS | -0.63 (-0.98, -0.33) | -0.16 (-0.36, 1.05) | 0.042 |
| FAH−TH (cm) | -6.00 (-7.40, -4.25) | 0.10 (-2.19, 1.38) | <0.001 |
| FAHSDS−THSDS | -0.32 (-0.70, 0.21) | 0.47 (0.37, 0.69) | 0.015 |
| FAHSDS−THSDS ≥ −1 | 17 (89.47%) | 8 (100.00%) | 0.9 |
| FAH−PAH (cm) | 2.66 (1.38, 4.74) | 7.62 (6.76, 9.24) | <0.001 |
| FAHSDS−PAHSDS | 1.27 (0.86, 1.68) | 1.80 (1.52, 2.07) | 0.015 |
| FAHSDS−PAHSDS ≥ 1 | 12 (63.16%) | 8 (100.00%) | 0.13 |
| FAH−Height(B) (cm) | 4.60 (3.40, 6.90) | 8.75 (7.58, 11.00) | 0.002 |
| FAHSDS−HtSDS | 0.00 (-0.27, 0.16) | 0.13 (-0.03, 0.49) | 0.3 |
| Note:Values are expressed as median (IQR).  *FAH* final adult height, *TH* target height, *SDS* standard deviation score, *FAHSDS−THSDS* the difference between final adult height SDS and target height SDS, *PAH* predicted adult height at baseline, *FAHSDS−PAHSDS* the difference between final adult height SDS and predicted adult height SDS at baseline,representing the benefit compared to the baseline predicted height, *Height (B)* height at baseline, *FAHSDS−HtSDS* the difference between final adult height SDS and baseline Ht SDS,representing the benefit of final adult height compared to baseline height | | | |

**Table S3. Multiple linear regression analysis for efficacy**

| Outcome | Variable | Coefficient (95% CI) | *P*-value |
| --- | --- | --- | --- |
| FAH−TH (cm) | Intercept | −29.571 (−48.102, −11.040) | 0.003 |
|  | Age (year) | 1.223 (0.125, 2.322) | 0.031 |
|  | HtSDS | 0.754 (−0.515, 2.024) | 0.230 |
|  | BA−CA (year) | 1.878 (0.440, 3.316) | 0.013 |
|  | rhGH dose (IU/kg) | 6.662 (−62.760, 76.083) | 0.843 |
|  | Duration of therapy (month) | 0.448 (0.286, 0.611) | <0.001 |
| FAHSDS−THSDS | Intercept | 1.186 (−2.703, 5.075) | 0.532 |
|  | Age (year) | −0.096 (−0.326, 0.135) | 0.397 |
|  | HtSDS | 0.050 (−0.217, 0.316) | 0.701 |
|  | BA−CA (year) | 0.352 (0.050, 0.653) | 0.025 |
|  | rhGH dose (IU/kg) | −6.012 (−20.582, 8.557) | 0.400 |
|  | Duration of therapy (month) | 0.049 (0.015, 0.083) | 0.007 |
| FAH−PAH (cm) | Intercept | −13.668 (−27.409, 0.072) | 0.051 |
|  | Age (year) | 1.024 (0.209, 1.839) | 0.016 |
|  | HtSDS | 0.841 (−0.100, 1.783) | 0.077 |
|  | BA−CA (year) | 0.900 (−0.166, 1.966) | 0.093 |
|  | rhGH dose (IU/kg) | −3.233 (−54.707, 48.241) | 0.897 |
|  | Duration of therapy (month) | 0.399 (0.278, 0.519) | <0.001 |
| FAHSDS−PAHSDS | Intercept | 4.702 (2.413, 6.991) | <0.001 |
|  | Age (year) | −0.197 (−0.333, −0.062) | 0.007 |
|  | HtSDS | 0.026 (−0.131, 0.183) | 0.732 |
|  | BA−CA (year) | 0.165 (−0.013, 0.343) | 0.067 |
|  | rhGH dose (IU/kg) | −7.165 (−15.740, 1.410) | 0.097 |
|  | Duration of therapy (month) | 0.041 (0.021, 0.061) | <0.001 |
| FAH−Height (B)(cm) | Intercept | 3.205 (−12.214, 18.624) | 0.669 |
|  | Age (year) | 0.306 (−0.609, 1.220) | 0.494 |
|  | HtSDS | 1.640 (0.584, 2.697) | 0.004 |
|  | BA−CA (year) | −0.523 (−1.719, 0.673) | 0.373 |
|  | rhGH dose (IU/kg) | −14.067 (−71.830, 43.696) | 0.617 |
|  | Duration of therapy (month) | 0.347 (0.212, 0.482) | <0.001 |
| FAHSDS−HtSDS | Intercept | −1.239 (−2.979, 0.500) | 0.153 |
|  | Age (year) | 0.060 (−0.043, 0.163) | 0.238 |
|  | HtSDS | −0.216 (−0.335, −0.096) | 0.001 |
|  | BA−CA (year) | −0.093 (−0.228, 0.042) | 0.165 |
|  | rhGH dose (IU/kg) | 0.945 (−5.571, 7.462) | 0.765 |
|  | Duration of therapy (month) | 0.041 (0.026, 0.056) | <0.001 |
| *BA* bone age, *CA* chronological age, *FAH* final adult height, *TH* target height, *SDS* standard deviation score, *HtSDS* height standard deviation score at baseline, *PAH* predicted adult height, *Height (B)* height at baseline, *rhGH* recombinant human growth hormone | | | |

**Table S4. Comparison of outcomes between groups and ANCOVA**

| Characteristic | Control  N = 12 | rhGH  N = 27 | Difference | 95% CI | *P* | Adjusted *P*-value† |
| --- | --- | --- | --- | --- | --- | --- |
| Dose of rhGH during treatment |  |  |  |  |  |  |
| Mean ± SD | - | 0.19 ± 0.01 | - | - | - |  |
| Median (IQR) | - | 0.19 (0.18, 0.20) | - | - | - |  |
| FAH (cm) |  |  |  |  |  | 0.051 |
| Mean ± SD | 155.58 ± 7.80 | 164.55 ± 8.23 | −8.97 | −14.68, −3.26 | - |  |
| Median (IQR) | 153.50  (150.00, 160.88) | 165.00  (161.35, 170.00) | - | - | 0.005 |  |
| FAHSDS |  |  |  |  |  | <0.001 |
| Mean ± SD | −1.28 ± 0.96 | −0.32 ± 0.87 | −0.96 | −1.63, −0.28 | - |  |
| Median (IQR) | −1.29  (−1.97, −0.55) | −0.54  (−0.78, −0.19) | - | - | 0.01 |  |
| FAH−TH (cm) |  |  |  |  |  | 0.097 |
| Mean ± SD | −7.08 ± 2.58 | −4.32 ± 3.69 | −2.76 | −4.87, −0.66 | - |  |
| Median (IQR) | −7.00  (−8.90, −5.38) | −4.80  (−6.40, −2.18) | - | - | 0.023 |  |
| FAHSDS−THSDS |  |  |  |  |  | 0.020 |
| Mean ± SD | −0.70 ± 0.60 | −0.05 ± 0.72 | −0.65 | −1.11, −0.19 | - |  |
| Median (IQR) | −0.88  (−1.12, −0.42) | −0.16  (−0.66, 0.51) | - | - | 0.006 |  |
| FAHSDS−THSDS ≥ −1 | 8 (66.67%) | 25 (92.59%) | −0.26 | −0.60, 0.09 |  |  |
| FAH−PAH (cm) |  |  |  |  |  | 0.013 |
| Mean ± SD | 0.82 ± 1.76 | 4.50 ± 3.04 | −3.69 | −5.26, −2.11 | - |  |
| Median (IQR) | 0.36 (−0.67, 2.09) | 4.52 (2.12, 6.40) | - | - | <0.001 |  |
| FAHSDS−PAHSDS |  |  |  |  |  | <0.001 |
| Mean ± SD | 0.69 ± 0.50 | 1.44 ± 0.55 | −0.75 | −1.11, −0.38 | - |  |
| Median (IQR) | 0.67 (0.31, 0.85) | 1.48 (1.04, 1.80) | - | - | <0.001 |  |
| FAHSDS− PAHSDS (B) ≥ 1 | 2 (16.67%) | 20 (74.07%) | −0.57 | −0.90, −0.25 | - |  |
| FAH−Height (B) (cm) |  |  |  |  |  | 0.016 |
| Mean ± SD | 2.44 ± 2.19 | 6.25 ± 3.06 | −3.81 | −5.58, −2.04 | - |  |
| Median (IQR) | 2.40 (0.33, 4.28) | 5.70 (3.50, 7.85) | - | - | 0.001 |  |
| FAHSDS−HtSDS |  |  |  |  |  | <0.001 |
| Mean ± SD | −0.28 ± 0.43 | 0.01 ± 0.48 | −0.29 | −0.61, 0.03 | - |  |
| Median (IQR) | −0.44  (−0.61, −0.02) | 0.01  (−0.23, 0.19) | - | - | 0.086 |  |
| Note:Values are expressed as median (IQR) or mean ± SD.  *FAH* final adult height, *TH* target height, *SDS* standard deviation score, *FAHSDS−THSDS* the difference between final adult height SDS and target height SDS, *PAH* predicted adult height at baseline, *FAHSDS−PAHSDS* the difference between final adult height SDS and predicted adult height SDS at baseline,representing the benefit compared to the baseline predicted height, *Height(B)* height at baseline, *FAHSDS−HtSDS* the difference between final adult height SDS and baseline Ht SDS,representing the benefit of final adult height compared to baseline height  † ANCOVA-adjusted *P*-value, with adjustment for variables including gender, baseline chronological age, and baseline height SDS. | | | | | | |
